# National Library of Medicine Disaster Information Management Research Center: Establishment and growth, 2008–2010 ^1^

**DOI:** 10.3233/ISU-130709

**Published:** 2013-12-02

**Authors:** Cynthia B. Love, Stacey J. Arnesen, Steven J. Phillips

**Affiliations:** a Disaster Information Management Research Center, Specialized Information Services Division, National Library of Medicine, Bethesda, MD, USA; b Specialized Information Services Division, National Library of Medicine, Bethesda, MD, USA

**Keywords:** National Library of Medicine, Disaster Information Management Research Center, history, preparedness, response, recovery, public health emergencies, emergency management, libraries, librarians, informationists, Bethesda Hospitals’ Emergency Preparedness Partnership, hazards databases, internet, web, research and development, WISER, REMM, CHEMM, Resource Guide for Public Health Preparedness, National Network of Libraries of Medicine

## Abstract

In 2008, the National Library of Medicine (NLM) established the Disaster Information Management Research Center (DIMRC). Prior to 2008, NLM had a long history of involvement in providing health information for disaster management. Aware of this legacy and moved by the catastrophic aftermath of Hurricane Katrina, the NLM long range plan (*Charting a Course for the 21st Century: NLM’s Long Range Plan 2006–2016*) called for creation of a center to show “a strong commitment to disaster remediation and to provide a platform for demonstrating how libraries and librarians can be part of the solution to this national problem”. NLM was urged to “ensure continuous access to health information and effective use of libraries and librarians when disasters occur”. In response to this charge, NLM has undertaken substantial efforts to ensure that medical libraries have plans for the continuity of their operations, librarians are trained to understand their roles in preparedness and response, online disaster health information resources are available for many audiences and in multiple formats, and research is conducted on tools to enhance the exchange of critical information during and following disasters. This paper documents the history, goals, initiatives, accomplishments and future plans of the Center.

## 1. Executive summary

The purpose of this paper is to review the history, objectives, initiatives, and future plans for the Disaster Information Management Research Center at the National Library of Medicine (NLM). Recognizing the untapped potential of libraries, librarians, and information services to aid in the nation’s disaster management efforts, the National Library of Medicine Board of Regents’ Long Range Plan (2006–2016) Subcommittee recommended that NLM create a Disaster Information Management Research Center (DIMRC) to support national emergency preparedness and response efforts. DIMRC opened in July 2008 with a staff of seven and is part of the NLM Specialized Information Services Division in Bethesda, Maryland.

NLM, now one of the National Institutes of Health and part of the U.S. Department of Health and Human Services, has played a pivotal role in translating biomedical research into practice since 1836. NLM, with nearly 12 million books, journals, manuscripts, audiovisuals and other forms of medical information, is available to people worldwide via resources such as PubMed and PubMed Central (references and full-text journal literature) for professionals and MedlinePlus health information for the public. As a research institute, performing and funding biomedical informatics research is also a major function of NLM.

In addition, NLM has a long history of providing health information, training and tools in response to all-hazards disasters. The National Library of Medicine encourages greater recognition of information management and access to health information resources as key components of disaster medicine and public health. The establishment of the Disaster Information Management Research Center reflects NLM’s commitment to this national priority. Work to date has made significant strides in the collection and dissemination of disaster health information, making it more readily available to first responders, health professionals and the public.

DIMRC coordinates all of NLM disaster-related activities, including:

- Collection, organization and dissemination of health information for all stages of preparedness and response to natural, accidental or deliberate disasters.
- Planning and training for continuity of operations at NLM, its eight Regional Medical Libraries, and the 5,800 member-libraries of its National Network of Libraries of Medicine.
- Training and support for librarians to act as Disaster Information Specialists in meeting their communities’ needs.
- Development of disaster and emergency health online resources for health professionals and the public.
- Raising awareness of NLM’s disaster health resources and providing training on their use.
- Disaster-related informatics research and development projects both at NLM and through grants to other institutions.
- Collaboration with other government agencies involved in disaster health and medicine to ensure information needs receive adequate attention in planning for disasters and in providing education and training for responders.

DIMRC’s Web site provides information about DIMRC and outlines current resources for disaster and emergency personnel including:

- Emergency Response and Toxicology Tools
  - WISER, the Wireless Information System for Emergency Responders.
  - REMM, the Radiation Event Medical Management System.
  - CHEMM, Chemical Hazard Emergency Medical Management. (This tool is under development.)
  - TOXNET, the Toxicology Data Network with over a dozen databases on hazardous chemicals.
- Subject Guides
  - Enviro-Health Links series with a dozen guides on disaster topics for health professionals.
  - MedlinePlus with nearly 40 subject pages on all-hazards topics for the public, in English and Spanish.
  - Partners in Information Access for the Public Health Workforce Web site.
- Disaster Medicine and Public Health Literature
  - PubMed with more than 40,000 medical journal articles on disaster topics from 5,000 journals, including 20+ journals exclusively on disaster and emergency medicine.
  - The NLM catalog with well over 1,000 publications on disaster topics related to medicine.
  - Resource Guide for Public Health Preparedness with links to over 3,000 online publications such as guidelines, reports, Web sites and fact sheets.

DIMRC plans to enhance and maintain its current resources and work with the disaster medicine and emergency management communities to develop new tools to better meet their critical information needs. DIMRC also coordinates outreach and training on the use of NLM resources by disaster personnel. Future activities include:

- Developing a comprehensive online resource for disseminating disaster health literature, building on NLM expertise in collecting and organizing biomedical information.
- Developing core competencies, curriculum, and certification (administered by an association or university) for Disaster Information Specialists and building libraries’ disaster readiness.
- Developing new tools to assist emergency responders in effectively and quickly responding to chemical, biological, radiological, and nuclear events.
- Continuing research and publishing results from current projects to improve hospitals’ communication systems redundancy, sharing of essential information, and tracking of patients during mass casualty events; updating and expanding a disaster informatics research agenda.
- Promoting NLM partnerships with hospitals and libraries as models for other geographic regions while continuing collaborations with government agencies and disaster medicine education and training programs.

Information is described as a key component in federal response to disasters by both Emergency Support Function #8 – Public Health and Medical Services of the National Response Framework and the Pandemic and All Hazards Preparedness Act. NLM has responded to the Senate request to create a Disaster Information Management Research Center to work with HHS and other lead agencies in disaster preparedness, response, mitigation and recovery. Sustained support for the concept of a Disaster Information Management Research Center will allow NLM to continue to enhance and improve its ability to develop tools and resources to provide disaster health information to emergency and disaster personnel, health professionals, and the public. Access to vital health information will help ensure that communities, individuals and government agencies will be better prepared for disaster response to any type of event and for resilient recovery.

## 2. Introduction

The purpose of this paper is to review the history, objectives, initiatives, and future plans for the Disaster Information Management Research Center (DIMRC) at the National Library of Medicine (NLM) [36]. This review may be of particular interest to NLM’s fellow sponsors of the Institute of Medicine’s Forum on Medical and Public Health Preparedness for Catastrophic Events [20]. Many of the Forum member institutions may both contribute to and benefit from effective DIMRC programs and Web resources. NLM looks to the Forum for guidance, support, and recommendations on priorities for information resources that will best serve those responsible for disaster medicine and public health preparedness, response and recovery.

The term “disaster” covers several types of events: natural disasters, technological disasters and acts of violence. Since 1953, there have been 1,858 declarations of disaster in the United States, according to the Federal Emergency Management Agency (FEMA), or an average of 30+ each year [10]. Many disasters present social and professional challenges to a community and its health workforce, including librarians. Catastrophic events overwhelm, at least temporarily, the ability of local or regional health systems to acquire information and deliver services consistent with established standards of care.

Recognizing the untapped potential of libraries, librarians, and information services to aid in the nation’s disaster management efforts, NLM Board of Regents’ Long Range Plan (2006–2016) [7] Subcommittee recommended that NLM create a “Disaster Information Management Research Center” to support national emergency preparedness and response efforts. DIMRC opened in July 2008 with a staff of seven and is part of the Specialized Information Services Division [55] in Bethesda, Maryland.

Senate Report 110–410 [11] on the 2009 appropriation bill for the Department of Health and Human Services included this statement about NLM and disaster health information:

The Committee supports the plan to establish a Disaster Information Management Research Center, which will lead Federal efforts to (A) identify and implement best practices for maintaining access to health information during disasters, (B) develop innovative products and services to serve emergency responders and preparedness activities, and (C) conduct research to support disaster health information management. The Center should also (D) collaborate with other Federal agencies, local communities and public health officials in efforts to prevent, respond to, and reduce the adverse health effects of disasters.

DIMRC coordinates all NLM disaster-related activities as outlined in the Senate Report excerpt above:

- Identify and implement best practices for maintaining access to health information during disasters.
  NLM is:
  - Collecting, organizing, and disseminating health information for all stages of preparedness and response to disasters.
  - Planning and training for continuity of operations at NLM, its eight Regional Medical Libraries, and the 5,800 member-libraries of its National Network of Libraries of Medicine (NN/LM) [38].
  - Training and supporting librarians to act as Disaster Information Specialists in meeting their communities’ needs.
- Develop innovative products and services to serve emergency responders and preparedness activities.
  NLM is:
  - Developing disaster and emergency health online resources for health professionals and the public.
  - Raising awareness of NLM’s disaster health resources and providing training on their use.
- Conduct research to support disaster health information management.
  - NLM is conducting disaster-related informatics research and development projects both at NLM and through grants to other institutions.
- Collaborate with other Federal agencies, local communities and public health officials in efforts to prevent, respond to, and reduce the adverse health effects of disasters.
  - NLM is collaborating with other government agencies involved in disaster health and medicine to ensure information needs receive adequate attention in planning for disasters and in providing education and training for responders.

DIMRC’s Web site [12] provides information about the Center, disaster health information resources, NLM emergency response and toxicology tools, medical and scientific literature, partnerships, research activities, and Disaster Information Specialist Project libraries and librarians.

## 3. History and background of National Library of Medicine disaster-related activities

NLM, now one of the National Institutes of Health and part of the U.S. Department of Health and Human Services, has played a pivotal role in translating biomedical research into practice since 1836. NLM, with nearly 12 million books, journals, manuscripts, audiovisuals, and other forms of medical information, is available to people worldwide via Web resources such as PubMed [46] and PubMed Central [47] references and full-text journal literature for professionals, and MedlinePlus [33] health information for the public. As a research institute, performing and funding biomedical informatics research is also a major function of NLM. Informatics research applies computer technology and communications to improve storage, retrieval, management and use of biomedical information.

The NLM commitment to providing disaster health information pre-dates the creation of the new Center by several decades. As far back as 1984, when the methyl isocyanate gas leak in Bhopal, India, galvanized public interest in industrial disasters, NLM was called on to provide crucial information from TOXNET, its Toxicology Data Network [57], which includes the Hazardous Substances Data Bank [22] comprehensive toxicological information on thousands of chemicals.

Following Hurricane Mitch in 1998, the White House and Congress requested federal agencies to provide assistance to the affected region in Central America. NLM responded by joining with the Pan American Health Organization to strengthen the local and national health information infrastructures in Central America, forming the Central American Network for Disaster and Health Information [6].

After the September 11, 2001, terrorist attack on the World Trade Center, the NLM National Center for Biotechnology Information (NCBI) [34] developed software to rapidly analyze poor-quality DNA samples and assist in identifying 9/11 victims’ remains [19]. OSIRIS, the Open Source Independent Review and Interpretation System [41], was also later used to identify victims from Hurricane Katrina.

NCBI and the National Institute of Allergy and Infectious Diseases developed the Influenza Virus Resource [26] in 2004 to provide genomic data on influenza strains for vaccine and infectious disease researchers. This resource presents data obtained from the Influenza Genome Sequencing Project [25] and GenBank [21], along with tools for flu sequence analysis. In addition, it provides links to other resources that contain flu sequences, publications and general information about flu viruses. As of November 2009, over 4,000 human and avian isolates have been completely sequenced and made available through GenBank.

Following Hurricane Katrina in 2005, medical and many other types of libraries were used by the public to access needed health information related to recovery and rebuilding efforts and to locate displaced relatives. Responder agencies also used libraries for communications when their own systems were unavailable. To assist emergency response and recovery efforts, NLM sent Personal Digital Assistant (PDA) devices with WISER (Wireless Information System for Emergency Responders) [60] installed to assist in identifying and responding to toxic chemicals in the disaster region. (Section 5 has more on WISER.) NLM also worked through its National Network of Libraries of Medicine to re-establish and maintain access to health information in the Katrina-affected region.

In 2007, NLM developed the Radiation Event Medical Management (REMM) [49] Web site, in collaboration with the HHS Office of the Assistant Secretary for Preparedness and Response (ASPR), the National Cancer Institute, and the Centers for Disease Control and Prevention (CDC). The REMM Web site provides guidance for health care professionals on diagnosis and treatment during mass casualty radiological or nuclear events. (Section 5 has more on REMM.)

NLM funded over 30 grants for disaster-related research from 2003 to 2007 through its Informatics for Disaster Management Awards [27] and Scalable Information Infrastructure Awards [52]. These awards supported pioneering work on automated biosurveillance, syndromic surveillance, and detectability of epidemics from over-the-counter sales. Research was also conducted on tracking patients during emergencies and on communications and scalable operations for disaster management.

As it has for nearly 175 years, NLM continues to collect and disseminate medical information through both its comprehensive online resources and its print, audiovisual and historical collections. (Appendix A has an inventory of current NLM disaster-related resources and programs.) For example:

- PubMed includes more than 40,000 medical journal articles on disaster topics from 5,000 journals, including 20+ journals exclusively on disaster and emergency medicine.
- MedlinePlus, a Web site for the public, has nearly 40 subject pages on disaster and all-hazards topics in both English and Spanish.
- The NLM catalog [37] lists well over 1,000 publications on disaster topics related to medicine, including nearly 150 on Hurricane Katrina and dozens on the health effects related to the dust, debris and clean-up following the September 11, 2001, World Trade Center terrorist attack and collapse, as well as publications on disasters occurring beyond U.S. borders.
- The TOXNET collection of databases on toxicology, hazardous chemicals, and environmental health provides comprehensive information on thousands of chemicals, maps of facilities releasing or containing toxic chemicals, and more. It’s an invaluable source for preventing and responding to hazardous materials incidents and the backbone of NLM tools for responders.
- The Enviro-Health Links [15] series has a dozen guides on disaster topics for health professionals, including a new page on the medical concerns following the Haiti earthquake.
- The Partners in Information Access for the Public Health Workforce [44] Web site covers bioter-rorism and public health preparedness.

## 4. About the Disaster Information Management Research Center

The Disaster Information Management Research Center became an official NIH “office” in July 2008, following a year and a half of planning. The staff includes seven information, computer and health professionals. DIMRC staff collaborate on projects with many others in their Specialized Information Services Division and with experts in informatics research, program evaluation, and collection development from other parts of NLM. DIMRC also uses contractor support for some activities.

DIMRC works with the NLM Board of Regents, senior staff, and its many partners to determine goals, priorities and users’ needs. DIMRC monitors and responds to current events such as the H1N1 influenza pandemic by selecting and disseminating current and authoritative sources of needed information. Through DIMRC’s activities, NLM is demonstrating how libraries and librarians can be part of the solution to national disaster response and is making a strong commitment to ensuring continuous access to health information when disasters occur.

The basic DIMRC budget is included within the Specialized Information Services Division funding through NLM appropriations. DIMRC also receives funding from ASPR for the production and maintenance of REMM and CHEMM. In FY2008 only, NLM also received funds from the Bethesda Hospitals’ Emergency Preparedness Partnership (BHEPP) [4] to conduct research and development projects related to emergency preparedness in hospitals.

## 5. DIMRC achievements and current initiatives

This review is based on the four goals identified in Senate Report 110–410 described in the Introduction.

### (A). Identify and implement best practices for maintaining access to health information during disasters

#### (A).1. NLM is collecting, organizing, and disseminating health information for all stages of preparedness and response to disasters

The collection, organization, and dissemination of pertinent disaster health literature are major DIMRC objectives. NLM has conducted several studies [9,58] investigating the information needs and information-seeking behaviors of emergency and disaster health professionals. Most study respondents indicated that disaster health literature was scattered and difficult to find and procure, and that a central location for this information was needed. Unlike many subjects in biomedicine, much of the disaster health information is not found in peer-reviewed journals indexed for PubMed or other databases. This “grey” literature, including reports from state and local health departments, evaluations, field assessments, after-action reports, lessons learned and guidelines, is diverse, not well organized or easy to find, and perishable.

NLM is investigating how to aggregate disaster health information from disparate sources for easy access. Key sources to consider for aggregating include PubMed, the NLM catalog, the Resource Guide for Public Health Preparedness [51], FEMA’s National Emergency Training Center Learning Resource Center [29], Lessons Learned Information Sharing [30] and the Homeland Security Digital Library [23]. Many of the available Internet sources that include disaster health information are limited either to particular types of documents or to narrow subject areas. Some are available only by password to users approved by the U.S. Department of Homeland Security. A recent review (Appendix B) of the disaster health content of more than 20 Web sites confirms the observation of study respondents that there is no single, dedicated resource for disaster medicine and public health information in all publication formats.

NLM is also working with several publishers to develop an Emergency Access Initiative plan to provide full-text access to key medical journals at no cost, following a widespread disaster that is expected to severely limit libraries’ abilities to function for at least several weeks. This initiative formalizes services from publishers that were spontaneously offered to medical libraries affected by Hurricane Katrina and will ensure they are available during future disasters. The first deployment of the Emergency Access Initiative is in support of medical relief efforts following the Haiti earthquake.

#### (A).2. NLM is planning and training for continuity of operations at its own facilities, its eight Regional Medical Libraries, and the 5,800 member-libraries of its National Network of Libraries of Medicine

As part of the National Institutes of Health, NLM follows the general Continuity of Operations Plan for NIH. NLM is also developing a more detailed plan for maintaining its own essential information services. NLM devotes considerable resources to ensuring uninterrupted access to its essential services in the event of a disaster or emergency, including back-up arrangements for its computing and communications systems.

NLM, through its National Network of Libraries of Medicine and its eight Regional Libraries, has contingency plans [2] to help ensure medical libraries will be able to offer services in the event of a disaster. Among the Regional Libraries, each has a partner to provide back-up assistance. The Regional Libraries, in turn, provide back-up and support for member libraries affected by local disasters. The National Network currently is focusing on disaster preparedness for medical and hospital libraries. Librarians throughout the United States have been trained on the “10-Step Approach to Service Continuity Planning” [53]. The NN/LM Emergency Preparedness & Response Toolkit [39] provides emergency contacts, sample forms, preservation information, and other sources to aid in disaster planning for maintaining library operations. The 2009 focus was on hospital libraries and included a national summit of hospital librarians sharing best practices for preparedness.

#### (A).3. NLM is training and supporting librarians to act as Disaster Information Specialists in meeting their communities’ needs

DIMRC is investigating the development of a new subject specialty role, the Disaster Information Specialist, to provide information and reference support for disaster medicine, public health prepared-ness, and emergency response. For a number of years, some medical libraries have offered the services of information specialists, or informationists, who provide research and knowledge management in a biomedical specialty for a clinical group or academic department. A Disaster Information Specialist would function in a similar role.

DIMRC provides support and training for librarians interested in disaster topics. Nearly 370 librarians subscribe to an e-mail forum that focuses on disaster health information outreach to communities and acts as a clearinghouse for sharing research, experiences, a weekly news digest and information requests. For example, the listserv has posted a dozen reviews of medical information to aid the Haiti earthquake relief efforts in the first two weeks following that disaster. Monthly conference calls with guest speakers build background knowledge and reinforce interests in disaster topics. By experimenting with roles for librarians and learning from experiences in previous disasters, this program will identify core competencies for a Disaster Information Specialist that will be used to design a national curriculum and develop a certification program administered by a major library association or information science program. Competencies in disaster health information will also be identified for emergency and disaster personnel for possible incorporation in their training programs.

To further develop the concept of the Disaster Information Specialist, DIMRC has pilot projects in four libraries (military, federal, academic, and community hospital) and one local medical library emergency preparedness partnership [31]. Projects underway are demonstrating that Disaster Information Specialists can contribute to disaster preparedness and response activities in their communities by acting as the information specialist for disaster personnel in their organizations. They can also analyze the information needs of emergency and disaster professionals, advise how NLM can meet those needs, and assist in the collection and dissemination of disaster health information.

### (B). Develop innovative products and services to serve emergency responders and preparedness activities

#### (B).1. NLM is developing disaster and emergency health online resources for health professionals and the public

NLM is both enriching its long-standing resources to provide appropriate coverage of disaster topics and also developing new tools and resources to aid in preparedness, response, and recovery. In the past few years, NLM has developed several tools to assist emergency personnel prepare for and respond to chemical, biological, radiological, and nuclear events and is investigating the use of new communication technologies.

WISER, the Wireless Information System for Emergency Responders, introduced in 2004, assists with the identification of and response to hazardous materials incidents involving chemical, biological or radiological agents. WISER is available for multiple platforms and can be downloaded to PDAs or computers using Windows, and also is available on the Web. WISER assists responders with quick identification of chemicals involved in an incident, and provides guidance on immediate, safe, and appropriate response. For each substance, it includes chemical and physical properties, human health effects, emergency medical treatment, personal protective equipment needs, and firefighting procedures. WISER has been widely adopted by the HAZMAT community, as reflected by the fact that it has nearly 34,000 subscribers.

REMM, the Radiation Event Medical Management Web site, was developed by NLM, ASPR, the National Cancer Institute and CDC. Since its release in 2007, NLM continues to host and enhance REMM. Project partners contribute new content and ensure that the site provides timely and accurate information. Extensive efforts to promote awareness of this resource are underway. REMM provides just-in-time, evidence-based information with sufficient background and context to make complex issues understandable to health care professionals who do not have formal radiation medicine expertise. REMM’s Web-based information is also downloadable to computers and mobile devices in advance, so that it can be available during an event if the Internet is not accessible.

In 2009, NLM and ASPR began collaborating on the Chemical Hazard Emergency Medical Management (CHEMM) Web site. CHEMM is being designed for use by emergency responders and health care providers who do not have hazardous materials training and are confronted with a large-scale chemical incident. CHEMM will focus specifically on chemicals that may be used in terrorist attacks to cause mass casualties and will provide links among chemical hazards information systems.

In addition to Web pages and databases published on the Internet, NLM is experimenting with the use of social media capabilities to facilitate communication, information sharing, interoperability and collaboration around disaster-related issues. Web sites such as Twitter, YouTube and Facebook provide platforms for citizens and responders to connect and exchange information, and their use and versatility continue to explode. In the most recent example, social media have been heavily used by many organizations and government agencies to inform the public about the H1N1 influenza outbreak.

Recognizing that building resources does not ensure their use, NLM widely promotes its disaster health-related tools and provides training on their use (as it does for all its resources). NLM staff, along with staff from the Regional Medical Libraries, present posters, papers, continuing education classes, and exhibits at disaster-oriented national and regional conferences. Training materials for online and classroom use are available. Tutorials, some providing Continuing Medical Education (CME) credit, are online. The disaster and emergency response community is reached through the Web sites, e-mail lists, journals, newsletters and other channels they most commonly turn to for professional development. Promotional materials are online, in print, and distributed on compact discs and flash drives. NLM reaches out directly to disaster personnel and also to the institutions (libraries, associations, universities) that support their information needs, education and training, and professional advancement.

### (C). Conduct research to support disaster health information management

#### (C).1. NLM is conducting disaster-related informatics research and development projects both at NLM and through grants to other institutions

Through grants, research contracts, and in-house activities, NLM supports emergency preparedness and response research and development projects. This research support is making a significant contribution to a field that has limited research funds (from any source) for disaster informatics and development of new technologies. As a research institute, NLM is committed to furthering the knowledge base and assisting in the development and evaluation of tools and resources to support emergency medical management. A small number of recent NLM Extramural Program research grants [50], applied informatics awards [28], small business awards [54], exploratory awards [17] and American Recovery and Reinvestment Act Awards [3], have relevance to disaster informatics.

A major focus for DIMRC in 2009 was coordination of the research agenda for the Bethesda Hospitals’ Emergency Preparedness Partnership. (Section 5.4 has more on this local partnership.) NLM joined BHEPP in 2008 to coordinate the partnership’s emergency preparedness and response research agenda. One-time funding of $3.5 million was provided to conduct research and develop prototypes for 11 projects currently underway. The projects fall into five categories: communications, patient information management, information access, family reunification, and hospital staff training in a virtual environment as shown in Table 1 and described in Appendix C.

Several projects, including the Digital Pen Recording of Triage Data, Patient Data Exchange, Lost Person Finder and the MARS (Military Affiliate Radio System) back-up communication system, were demonstrated and/or tested at the Collaborative Multi-Agency Exercise [16] in October 2009. A version of the Lost Person Finder that may be of use following the Haiti earthquake is in accelerated production.

NLM is also investigating natural language processing and semantic tools to analyze disaster-related information. A pilot study is investigating ways to more effectively search the Web to find connections among concepts not easily identified by traditional text word methodologies, such as searching the Resource Guide for Public Health Preparedness for semantic connections related to pandemic influenza.

### (D). Collaborate with other Federal agencies, local communities and public health officials in efforts to prevent, respond to, and reduce the adverse health effects of disasters

#### (D).1. NLM is collaborating with other government agencies involved in disaster health and medicine to ensure information needs receive adequate attention in planning for disasters and in providing education and training for responders

NLM works with a number of agencies and organizations to help research and meet disaster health information needs, and seeks to develop new working relationships with additional agencies. Several of these partners, including the Disaster Information Specialist partner institutions, ASPR, and CDC, have been described elsewhere in this paper. Through its partnerships, NLM advocates for the role of libraries, librarians, and the use of its own collections and online resources in meeting disaster health information needs. Examples of activities with international, federal, national, local, and educational institutions are highlighted below.

##### International partnerships

As noted in the History section above, NLM has worked with the Pan American Health Organization and the Regional Disaster Information Center for Latin America and the Caribbean [48] since 2000. NLM provides funding and technical support for six countries^2^ to maintain the Central American Network for Disaster and Health Information. These countries have established local disaster information centers to collect, organize, and disseminate public health and medical information related to disasters. These centers developed a digital library of over 12,000 full-text documents, many in Spanish, to assist planners and responders in their countries quickly access vital information that was previously unavailable. In addition, the South-East Asia Regional Office of the World Health Organization is considering the development of a similar network.

##### Federal partnerships

DIMRC has developed projects with other federal agencies to help meet their information needs and to improve access to disaster health information. For example, DIMRC is working with ASPR on several projects, including REMM, CHEMM, the Disaster Information Specialist (in conjunction with the NIH Library), and the inclusion of high resolution images of prescription pills from the Strategic National Stockpile [56] in the Pillbox [45] Web site. The NLM History of Medicine Division is working with ASPR on collecting documents and oral interviews about the federal response to the 2009 H1N1 influenza outbreak. NLM partners with the Department of Transportation (DOT) to include the *Emergency Response Guidebook* [13] in WISER, and DOT, in turn, includes WISER with the *Emergency Response Guidebook* compact disc that is mailed to emergency responders across the country. This sharing of information and data has improved the usefulness of both of these resources for the emergency response community. NLM also participates in the Federal Education and Training Interagency Group and serves on the NIH Biodefense Research Coordinating Committee. In developing REMM, CHEMM and other tools, NLM works with CDC, the National Cancer Institute, the Environmental Protection Agency and other agencies.

##### National partnerships

NLM leads major, national partnerships and is also an active partner in national groups such as the Institute of Medicine’s Forum on Medical and Public Health Preparedness for Catastrophic Events.

The National Network of Libraries of Medicine, an NLM program, has eight Regional Medical Libraries and 5,800 member-libraries in all parts of the United States. The Network is NLM’s field force for outreach to health workers, training on NLM information resources, funding of local and regional projects, and coordination of medical library services. To improve disaster preparedness, NN/LM provides outreach and training on disaster information resources for librarians, emergency and disaster personnel, health professionals, and the public. During and after an event, network libraries are ready to meet the information needs of local responders and have the capacity to shift their workloads in response to loss of local or regional ability to provide library services. Regional Medical Libraries have been very active in reaching out to their members following events such as Hurricane Katrina, Hurricane Rita, Hurricane Ike and the 2009 Fargo, North Dakota flooding.^3^

NLM supports the Partners in Information Access for the Public Health Workforce project and hosts its Web site. Through this project, NLM has ties with 13 participating federal public health agencies and major associations representing the public health workforce [1]. The project promotes informatics and information skills for the public health workforce, as described in many of the *Core Competencies for Public Health Professionals* [8]. Strengthening information dissemination for public health workers, promoting information skills, and maintaining this coalition of supporters of public health information infrastructure improves capacity for meeting public health challenges, including the capacity for disaster preparedness and response.

##### Local partnership

NLM participates in a unique partnership to coordinate local hospital preparedness. In 2004, the NIH Clinical Center, the National Naval Medical Center, and Suburban Hospital/Johns Hop-kins Medicine established the Bethesda Hospitals’ Emergency Preparedness Partnership. NLM joined in 2008. A recent video dramatizes the Partnership’s efforts [5]. The Partnership leverages its complementary resources to prepare for and respond to emergencies in the National Capital Region and to conduct research related to emergency preparedness and response. BHEPP was funded by a Congressional earmark from FY2005 to 2008, with no continuance of funding for 2009–2010. BHEPP activities include purchase of shared equipment and supplies for increased medical surge capacity, strengthening of communications interoperability, addressing potential transportation and water vulnerabilities, cross-credentialing of hospital staff, conducting hospital emergency response research and conducting drills. (Section 5.3 has descriptions of BHEPP research projects conducted by NLM since 2008.)

##### Educational institutions

NLM is also involved in education and training efforts in public health in-formatics and in medical and public health disaster preparedness and response. In 2009, NLM and the Uniformed Services University of the Health Sciences collaborated on a one-semester Public Health Informatics course [59] built around the topic of all-hazards preparedness and response. The course featured the latest disaster-related information and online tools, use of social media and Web 2.0 tools, emerging informatics technologies and disease surveillance.

NLM serves on the Federal Education and Training Interagency Group established by Homeland Security Presidential Directive 21: Public Health and Medical Preparedness.^4^ The Group is fostering the development of the new National Center for Disaster Medicine and Public Health [35] at the Uniformed Services University. The Center will determine core competencies and education and training standards across federal, state, and local government, academia, and the private sector.

## 6. Next steps and plans for 2010–2012

The National Library of Medicine encourages greater recognition of information management and access to health information resources as key components of disaster medicine and public health. The establishment of the Disaster Information Management Research Center reflects NLM’s commitment to this national priority. Work to date has made significant strides in the collection and dissemination of disaster health information, making it more readily available to first responders, health professionals, and the public.

DIMRC plans to enhance and maintain its current resources and to expand its work with the disaster medicine and emergency management communities to develop new resources to better meet their critical information needs. These plans, based on the goals in Senate Report 110–410, are outlined as follows:

- Identify and implement best practices for maintaining access to health information during disasters.
  - Develop an online resource for disseminating disaster health literature, building on NLM expertise in collecting and organizing biomedical information.
  - Identify core competencies of a Disaster Information Specialist, design a national curriculum, and develop a certification program administered by a major library association or information science program at a university.
  - Evaluate and publish results from the Disaster Information Specialist Pilot Projects conducted at four libraries and fund new projects for libraries in partnership with disaster professionals.
  - Complete and widely promote the Emergency Access Initiative.
  - Continue training for librarians on the “10-Step Approach to Service Continuity Planning” and fully integrate disaster planning into routine library administration practices among the 5,800 member-libraries of the National Network of Libraries of Medicine.
  - Update and maintain a detailed plan for NLM continuity of operations of its own essential information services.
- Develop innovative products and services to serve emergency responders and preparedness activities.
  - Complete development and public release of the CHEMM Web site.
  - Develop additional content for REMM and promote awareness of this resource among health care professionals and librarians.
  - Enhance WISER with additional biological agents and develop an iPhone application and Blackberry-compatible version.
  - Continue experiments with the use of social media to facilitate routine exchanges about disaster information and literature, and also to serve as a communications tool during disasters.
  - Continue activities that raise awareness of NLM’s disaster health resources and provide training on their use, matching appropriate resources to the right target audiences for “just what I need, just in time” information.
- Conduct research to support disaster health information management.
  - Complete the initial scope of research for the BHEPP projects, and evaluate and publish the results.
  - Identify partners and resources to continue selected BHEPP research.
  - Transition BHEPP research prototypes to operational systems to be used by hospitals during drills and disasters.
  - Fund new and continuing disaster-related informatics research through grants administered by NLM Extramural Programs.
  - Build on current research at the NLM Lister Hill National Center for Biomedical Communications [32] and Office of Computer and Communications Systems [40] to expand their disaster-related projects.
- Collaborate with other Federal agencies, local communities and public health officials in efforts to prevent, respond to, and reduce the adverse health effects of disasters.
  - Promote BHEPP as a model for local hospital partnerships in other communities.
  - Promote the Central American Network for Disaster and Health Information as a model for other regions.
  - Coordinate with disaster medicine and public health training and education efforts underway at the Uniformed Services University of the Health Sciences National Center for Disaster Medicine and Public Health and other federal agencies.
  - Investigate the role NLM can play in developing a network among existing fusion centers and visualization laboratories.

Information is described as a key component in federal response to disasters by both Emergency Support Function #8 – Public Health and Medical Services of the National Response Framework [14] and the Pandemic and All Hazards Preparedness Act [43]. NLM has responded to the Senate request to create a Disaster Information Management Research Center to work with HHS and other lead agencies in disaster preparedness, response, mitigation and recovery. Sustained support for the concept of a Disaster Information Management Research Center will allow NLM to continue to enhance and improve its ability to develop tools and resources to provide disaster health information to emergency and disaster personnel, health professionals, and the public. Access to vital health information will help ensure that communities, individuals and government agencies will be better prepared for disaster response to any type of event and for resilient recovery.

## Figures and Tables

**Table 1:** Bethesda Hospitals’ Emergency Preparedness Partnership research and development projects

Communications	Patient information management	Information access	Family reunification	Hospital staff training
Laser communications between hospitals	Digital pen recording of triage data	SureScripts-Rx Hub prescription data access	Lost Person Finder	Virtual world hospital incident command system training
Dark fiber communications between hospitals	Patient data exchange among hospitals	Disaster Information Specialist		
MARS radio for digital communication	Tracking patients with RFIDs			
Wireless communications bridge				

## Footnotes

2 The six countries are Guatemala, Costa Rica, Panama, Honduras, Nicaragua and El Salvador.

3 Each network office has similar blogs with news entries for disasters in their region [18].

4 See [24], the Federal Education and Training Interagency Group is described in Paragraph 37. The National Center for Disaster Medicine and Public Health is in Paragraph 38.

## Appendix A. A–Z of disaster-related projects and resources from the National Library of Medicine^5^

### Bethesda Hospitals’ Emergency Preparedness Partnership (BHEPP)

NLM manages the BHEPP research and development projects portfolio as a member of this partnership, http://bhepp.org. Appendix C has a list of the projects.

### Central American Network for Disaster and Health Information (CANDHI)

NLM supports 10 Disaster Information Centers as part of the CANDHI network, http://www.relaciger.org/, in six Central American countries. These centers, along with the Regional Disaster Information Center for Latin America and the Caribbean, collect, organize, digitize and disseminate information pertaining to disasters in their locales.

### CHEMM – Chemical Event Medical Management

NLM recently began working on a tool, similar to Radiation Event Medical Management (REMM), for use by those who do not have hazardous materials training and are confronted with a large-scale chemical incident.

### Continuity of Operations Planning (COOP)

In addition to following the Continuity of Operations Plan for NIH, NLM has a detailed plan for maintaining its own essential services in the event of a prolonged emergency or an infectious disease outbreak that affects staff ability to report to work. The National Network of Libraries of Medicine and its eight Regional Libraries also have contingency plans for the network and its 5,800 members.

### DIRLINE

This Directory of Health Organizations, http://dirline.nlm.nih.gov, includes nearly 700 state government agencies that have disaster-related responsibilities, such as homeland security, property insurance regulation, health and mental health services, and public safety departments. An additional 8,000 organizations cover a wide range of health topics.

### Disaster Information Management Research Center

The Center is responsible for coordinating NLM’s disaster-related activities. It houses eight staff in Bethesda, Maryland, and includes equipment, software, and communications to explore new technologies related to disaster information and communication management, including visualization tools, virtual worlds, and radio communications. The Center Web site, http://disasterinfo.nlm.nih.gov, highlights NLM disaster-related activities and resources.

### Disaster Information Specialist Project

This project supports librarians who provide information outreach to their institutions and communities before, during, and after disasters. Four libraries and a partnership are pilot participants in experimenting with roles librarians can play in a disaster. Monthly conference calls highlight libraries’ disaster outreach activities.

### Disaster terminology

Medical Subject Headings (MeSH) are used to index journal articles and other publications at NLM. This page, http://sis.nlm.nih.gov/dimrc/glossaries.html, points to all the MeSH disaster terms and lists 60+ glossaries from other sources. NLM is funding the World Association of Disaster and Emergency Medicine to work on standardization of disaster medicine terminology.

### DISASTR-OUTREACH-LIB Listserv

Nearly 350 librarians subscribe to this NLM e-mail forum that acts as a clearinghouse for sharing research, experiences, a weekly news digest and information requests with a focus on disaster health information outreach to communities.

### Enviro-Health Links

This series includes a dozen guides on disaster topics for a professional audience, including biological and chemical threat agents, hurricanes, and tornadoes. A complete list can be found at http://disaster.nlm.nih.gov/dimrc/subjectguides.html. The newest addition is a page on the Haiti earthquake.

### Emergency Access Initiative

The Emergency Access Initiative (EAI) is designed to provide full-text access to key medical journals at no cost, following a widespread disaster that is expected to severely limit libraries’ abilities to function for at least several weeks. EAI is a partnership of NLM and the Professional/Scholarly Publishing Division of the Association of American Publishers.

### Grants and research contracts

NLM has funded a variety of disaster-related grants and contracts, including research on disaster management informatics and scalable information infrastructure. More information can be found at http://disasterinfo.nlm.nih.gov/dimrc/fundedresearchnlm.html.

### Influenza Virus Resource

This resource presents data obtained from the Influenza Genome Sequencing Project and GenBank, along with tools for flu sequence analysis. It provides links to other resources that contain flu sequences, publications, and general information about flu viruses at http://www.ncbi.nlm.nih.gov/genomes/FLU.

### MedlinePlus

MedlinePlus, http://medlineplus.gov, includes nearly 40 topic pages on disaster and hazards topics for the public, in English and Spanish. A complete list of disaster-related MedlinePlus topics in English and Spanish is available at http://disasterinfo.nlm.nih.gov/dimrc/subjectguides.html.

### MedlinePlus Go Local

MedlinePlus English-language pages link to Go Local, http://www.nlm.nih.gov/medlineplus/golocal, to find health resources in a state or region. Some Go Local areas customize and update a disaster page when an event occurs. [2013 Update: MedlinePlus Go Local was discontinued in 2011.]

### NN/LM Emergency Preparedness & Response Toolkit

This National Network of Libraries of Medicine (NN/LM) Toolkit, http://nnlm.gov/ep, provides emergency contacts, sample forms, preservation information and other sources to support planning and response to disasters that affect library services. The Toolkit, along with training on the “10-Step Approach to Service Continuity Planning”, assists librarians in developing preparedness plans and continuing services during disaster recovery.

### NLM Catalog

The NLM catalog, http://www.ncbi.nlm.nih.gov/nlmcatalog, includes over 1,000 publications on disaster topics related to medicine. The catalog includes links to electronic-only Internet documents as well as print materials in the NLM collection.

### Partners in Information Access for the Public Health Workforce

This Web site, http://phpartners.org, covers all topics of interest to the public health workforce and is supported by several partners, including NLM. Specific to disasters, the site includes bioterrorism and public health preparedness.

### PubMed

PubMed, http://pubmed.gov, includes more than 40,000 medical journal articles on disaster topics from 5,000 journals, including 20+ journals exclusively on disaster and emergency medicine.

### Rapid Research Notes

The Rapid Research Notes site, http://www.ncbi.nlm.nih.gov/rrn/about, provides permanent access to research shared through online forums designed for immediate communication. Its first collection is a forum from the Public Library of Science, *PloS Currents: Influenza*, on 2009 H1N1 influenza research.

### REMM – Radiation Event Medical Management

REMM, http://www.remm.nlm.gov, provides guidance for health care professionals on diagnosis and treatment during mass casualty radiological or nuclear events such as dirty bombs, nuclear power plant accidents and nuclear explosions.

### Resource Guide for Public Health Preparedness

The New York Academy of Medicine Library, with NLM funding, built and maintains this Web site, http://phpreparedness.info, that links to over 3,000 online publications such as guidelines, reports, Web sites, and fact sheets for public health workers.

### TOXNET

The TOXNET collection of databases on toxicology, hazardous chemicals and environmental health, http://toxnet.nlm.nih.gov, provides comprehensive information on thousands of chemicals. It’s an invaluable source for preventing and responding to hazardous materials incidents and the backbone of NLM tools for responders.

### WISER – Wireless Information System for Emergency Responders

WISER, http://wiser.nlm.nih.gov, assists emergency personnel with hazardous materials incidents. It includes response information on over 400 chemicals, 20 radiological agents, and the CDC Class A biological agents as well as the full text of the *Emergency Response Guidebook* and the *WMD* [Weapons of Mass Destruction] *Response Guidebook*.

## Appendix B. Analysis of Web sites that collect disaster-related information: Health content, scope and ease of access^6^

In studies undertaken by NLM and others, the most frequent request by disaster planners, responders and health professionals is for a single Web resource that aggregates the medical and public health information they need for disaster training, preparedness, response and recovery. These studies, along with anecdotal feedback, identified many Web sources used by disaster professionals but none that were considered to be a single point-of-entry to the abundant available information.

To further assess the validity of the disaster professionals’ perceptions of Web resources, this analysis reviewed sites whose primary purpose is to collect disaster-related, technical, medical, or health information from a variety of resources. Also included are Web resources widely used to find Internet sites and information on a topic and the most popular medical Web sites. It does not include sites that primarily or exclusively feature their own publications. For example, many organizations such as the Centers for Disease Control and Prevention or the World Health Organization publish vital disaster health information but it is not part of their mission to also collect and disseminate materials from other organizations. The review includes selected, major hazards-related libraries and resource centers identified by the Natural Hazards Center Library [42].

This analysis confirms the disaster professionals’ observations that there is no single, dedicated Web site or database collecting disaster medicine and public health information in all formats. Internet sources with disaster health information are usually limited to particular types of documents or to specific subject areas. For example, a database may include journal article citations but not training videos or government reports. A Web site might focus on natural disasters but not bioterrorism. Some are available only by password to users approved by the U.S. Department of Homeland Security.

The appendix lists the reviewed Web sites with brief comments on their content and scope. “Limitations” are noted as they might compare to an ideal of an all-inclusive resource and not as an evaluation of the intended scope of a site. The list starts with Web sites that focus on disaster information and then reviews sites that collect health information. It ends with sites that may include some disaster health information although that is not their primary focus.

### B.1. Web sites aggregating disaster information

#### FEMA Learning Resource Center

http://www.lrc.fema.gov

##### Content

Provides current information and resources on fire and emergency management subjects. Contains over 100,000 books, periodicals, reports, and audiovisual materials, many of which can be obtained through interlibrary loan. Not to be confused with the “FEMA Library”, a database of FEMA publications at http://www.fema.gov/library.

##### Limitations

Scope is fire and emergency management, so health is covered as it relates to those topics. Not exhaustive in disaster health.

#### Homeland Security Digital Library (HSDL)

https://hsdl.org

##### Content

Homeland security-related documents collected from federal, state, tribal, and local government agencies, professional organizations, think tanks, academic institutions and international governing bodies.

##### Limitations

Password protected. Focus is primarily on homeland security, although some materials are related to disaster health.

#### Lawson Terrorism Information Center

https://resources.mipt.org/Resources/eDocuments.aspx

##### Content

Public Health section, http://www.terrorisminfo.mipt.org/Public-Health.asp, contains 212 reports, 9 fact sheets, 99 articles, 1 journal and 67 links (388 total).

##### Limitations

Emphasis is on terrorism, so health information is included when related to terrorism. Not a comprehensive health information resource, although many materials are included.

#### Lessons Learned Information Sharing

https://www.llis.dhs.gov/

##### Content

Documents related to homeland security and emergency response, including original research about lessons learned, best practices, practice notes and “good stories”. Also includes after-action reports; plans, procedures, templates and tools; and reports and documents.

##### Limitations

Password protected and access restricted to approved users. Includes limited health/public health materials. Can not link from other sites to original content written for the site such as lessons learned, good stories, etc. Balance of content is from publicly-accessible sources that here are limited to password-access only.

#### ReliefWeb

http://www.reliefweb.int

##### Content

Extensive news and bibliographic information on natural hazards and disaster-related events and human rights documents.

##### Limitations

Primary emphasis is on humanitarian crises, complex emergencies, and natural disasters. Not a resource for medical information. Heavily used by international relief agencies.

#### Resource Guide for Public Health Preparedness

http://www.phpreparedness.info

##### Content

Published by New York Academy of Medicine. Includes only freely available Web materials in electronic format. Uses subject headings developed specifically for the Resource Guide. See http://www.phpreparedness.info/help/SubjectHeadings.doc for headings, up to three of which are assigned to each document.

##### Limitations

Stronger on bioterrorism than on other disaster-related topics. Materials selected more for public health workers than other types of health workers. No subscription or print materials. Contains about 3,200 records.

### B.2. Selected examples of Web sites aggregating disaster information for a specific audience or subject

#### Avalanche Research Media Library

http://www.avalanche-research.com

##### Content

A collection of videos, along with some accident reports, photographs, maps, television shows, and audio files about avalanches. The site acts as a memory bank for the avalanche community on both a local and global scale. Users can browse the collection and submit their own videos and documents for the community. Includes search and rescue topics.

##### Limitations

Very narrow, but unique, in scope. New content added sporadically. Appears to be the work of a single individual.

#### Centers for Public Health Preparedness Resource Center

http://preparedness.asph.org/cphp/ResourceCenter.cfm

##### Content

Training and educational resources developed by the Centers for “reaching and teaching the public health workforce”.

##### Limitations

The Resource Center database includes only publications from the 30 member centers. Publication media types include webcasts, course outlines, CD-ROMs, DVDs, drills, games, and slide presentations.

#### Central American Network for Disaster and Health Information (CANDHI)

http://www.relaciger.org/

##### Content

Products, services, and other resources available on CANDHI in the region and beyond.

##### Limitations

Written primarily in Spanish. Central America focus.

#### FirstResponder.gov

http://www.firstresponder.gov

##### Content

Department of Homeland Security site provides access to anything and everything that might be of interest to first responders.

##### Limitations

Does not attempt to compile publications or resources, other than listing links to agencies and programs.

#### HazLit

http://ibs.colorado.edu/hazards/library/hazlit/NatHazSearch.php

##### Content

Catalog for the Natural Hazards Center Library collection which focuses on the social science literature related to natural hazards. Includes more than 2,200 records on public health or emergency medical services as related to natural disasters.

##### Limitations

Modest coverage of medical information related only to natural (not man-made) disasters. Catalog records do not include the full text of documents and the library does not lend from its collection.

#### Health Library for Disasters

http://helid.desastres.net/

##### Content

This library (HeLiD) includes 650 publications in English, Spanish and French on public health for disasters and complex emergencies. The collection is selected and hosted on a Web site by the Pan American Health Organization (PAHO). The full-text documents are primarily from major international health organizations.

##### Limitations

This library is a static collection of documents that is updated every few years and was last updated in 2007.

#### Public Health Systems and Preparedness Database

http://www.rand.org/health/centers/public-health-systems-and-preparedness.html

##### Content

Drills, tabletop exercises, and similar materials collected by RAND from around the country.

##### Limitations

Only about 100 exercises used to evaluate public health preparedness. No other types of documents or subject areas. Narrow scope. Project ended in 2005.

#### Toolkits from the National Association of County and City Health Officials (NACCHO): EQUIPh, Medical Reserve Corps, STOCKbox and H1N1

http://www.naccho.org/toolbox

##### Content

EQUIPh: Public health preparedness tools and resources from many program and topic areas for local health departments. Medical Reserve Corps: NACCHO products and tools developed by health agencies of use to Medical Reserve Corps. STOCKbox: Items related to Strategic National Stockpile.

##### Limitations

Many documents can be viewed only if user is registered. Registration is free. Documents are tools for departments to use, such as forms, assessments, and program guides. Scope is limited to public health and types of documents (toolkits). EQUIPh Toolkit has 180 records; Medical Reserve Corps Toolkit has 63; STOCKbox Toolkit has 103 and H1N1 Toolkit has 103.

#### TrainingFinder Real-Time Affiliate Integrated Network (TRAIN)

http://www.train.org

##### Content

Resource from the Public Health Foundation for finding public health training courses primarily taught online. 25 states participate.

##### Limitations

Requires free registration. Narrow in type of material, containing only training courses. Includes all public health topics.

#### United Nations International Strategy for Disaster Reduction (ISDR) Library on Disaster Reduction

http://www.unisdr.org/eng/library/lib-index.htm.

##### Content

The ISDR Library maintains a specialized collection focused on disaster risk reduction and related issues, including sustainable development, climate change, warning systems, environment, education, and gender.

##### Limitations

Less than 5% of the collection is on the medical and public health aspects of disaster reduction.

### B.3. Web resources aggregating medical and health information on all topics

#### BioMed Central

http://www.biomedcentral.com

##### Content

Repository for free full-text research articles from participating journals. Some of the 158 titles are specifically in Emergency Medicine, Public Health.

##### Limitations

Site has index to medical information only in the 158 member titles. Journal content other than research articles from some participating journals may not be included in BioMed Central. Registration required to search content.

#### Grey Literature Report

http://www.greylit.org/

##### Content

Items added to the New York Academy of Medicine (NYAM) library catalog under heading “grey literature”. The literature is from selected publishers: http://www.greylit.org/publishers/list. To see grey lit items, search on the Quick Limit “Grey Literature”.

##### Limitations

Features a broad focus on medicine, not specifically disaster-related. Includes only items that meet the NYAM Library’s selection criteria. Over 12,000 grey literature items in catalog. Links to electronic documents are not updated. NYAM has a print copy of each item.

#### MedlinePlus

http://medlineplus.gov

##### Content

Extensive content for consumers on many disaster-related topics. Selective, quality-controlled content.

##### Limitations

Includes only documents that are Web-accessible and only consumer-level documents. No archiving of older documents.

#### NLM Catalog

http://locatorplus.gov/cgi-bin/Pwebrecon.cgi?DB=local&PAGE=First http://www.ncbi.nlm.nih.gov/nlmcatalog

##### Content

Bibliographic description of materials in all formats selected for the NLM collection. Includes grey literature electronic documents with links updated when needed.

##### Limitations

Site is not a resource the average information seeker would know about or think to consult.

#### PubMed

http://pubmed.gov

##### Content

Comprehensive, free index to peer-reviewed medical literature, including all disaster-related topics.

##### Limitations

Includes only journal articles and abstracts.

#### PubMed Central

http://www.pubmedcentral.nih.gov

##### Content

Repository of free full-text articles from over 650 participating journals.

##### Limitations

Includes only publishers who choose to participate. Can limit searches in PubMed to only PubMed Central journals. PubMed (not PubMed Central) is the index to the medical literature.

### B.4. Federal government technical information Web sites on all topics

#### National Technical Information Service (NTIS)

http://www.ntis.gov

##### Content

Some disaster health content, but focus is on the type of material, i.e., reports, not subjects.

##### Limitations

Contains only government reports deposited by federal agencies. There is no subject index. NTIS charges for all materials as it is a clearinghouse for sales of reports.

#### Defense Technical Information Center

http://www.dtic.mil

##### Content

Exhaustive range of topics with military emphasis.

##### Limitations

Some materials require security clearance for access. Health materials included only as they relate to the military. Good source for finding materials but not a resource for a wide range of disaster health info.

### B.5. Popular, commercial medical information Web sites

#### EverydayHealth.com

http://www.everydayhealth.com

##### Content

Popular, heavily promoted consumer health information Web site that merged with Revolution Health.

##### Limitations

Site is commercial, contains many ads, and has very little information on disaster topics. The site has a swine flu center.

#### WebMD

http://www.webmd.com

##### Content

Materials for consumers, many from.gov sites.

##### Limitations

No clear section or topic heading that pulls together the wide array of consumer news, videos, reprints, and other materials on the disaster topics it offers for consumers. Much of the disaster content is repeated from.gov sites and is thus already on MedlinePlus. There is a topic page for H1N1 influenza.

### B.6. Internet searching

#### Google

http://google.com

##### Content

Most popular search engine for accessing the Internet. The first place most people go.

##### Limitations

Usually can not retrieve materials that reside in a database with its own search engine, unless the database has set up an index for Google crawling. Exhaustive results. No access point just for disaster topics. No selection criteria or quality filter.

## Appendix C. Research and development projects for the Bethesda Hospitals’ Emergency Preparedness Partnership^7^

The National Library of Medicine joined the Bethesda Hospitals’ Emergency Preparedness Partnership (BHEPP) in 2008 to coordinate the Partnership’s emergency preparedness and response research agenda. One-time funding of $3.5 million was provided to the Partnership to conduct research and develop prototypes for 11 projects started in 2009 and concluding in 2010. The research supports the Partnership’s efforts to enhance and sustain effective disaster response by evaluating innovative methods of sustaining communications during hospital disaster operations and ensuring essential information is accessible to hospital personnel at the point of need in a disaster. The Partnership Web site, http://www.bhepp.org, includes reports, photos and video about the projects.

### C.1. Communications

#### Laser communications between hospitals

Roof-top lasers will be installed at each partner hospital and will transmit data between the hospitals as a back-up, dedicated disaster communications system.

#### Dark fiber communications between hospitals

A dedicated optical fiber network is established which will be used to transmit data between the hospital partners as a back-up method of disaster communications.

#### MARS radio back-up

The Military Affiliate Radio System (MARS), a Department of Defense-sponsored entity, will be evaluated as a back-up communications system by moving digital information via radio in a disaster.

#### Wireless communications bridge

Technology will be evaluated that links voice communications systems used in disaster response including hand-held devices, land-line phone, mobile phone and radio, permitting cross-device communications.

### C.2. Patient management

#### Digital pen recording of triage data

Will evaluate the use of digital pens that record disaster patient triage data and uplink the data to a computer database; will preserve data and provide hospital personnel with overview of patient status.

#### Tracking patients with RFID

Will evaluate tracking disaster patients and critical hospital equipment; only research project not yet begun due to funding issues still being resolved.

#### Patient data exchange

A core set of patient data elements is being devised so that hospital partners can communicate common patient data in a disaster.

### C.3. Family reunification in disaster

#### Lost Person Finder

Will use information technology to facilitate reunification of separated people; will permit them to locate the missing via computer, mobile phone and other media. Will enhance situational awareness and response as well for hospital and emergency management professionals dealing with family reunification issues.

### C.4. Hospital staff training

#### Virtual World Interactive Disaster Training

An online ‘virtual’ hospital environment will be constructed to permit hospital staff to train for disaster response in a realistic manner.

### C.5. Information access

#### SureScripts-Rx Hub prescription data access

Will evaluate a system to access patient medication data from commercial databases to provide clinicians with critical data that influences clinical decision-making in disaster and is often unavailable.

#### The Disaster Information Specialist

Will evaluate the innovative use of hospital librarians as disaster information resources available to support clinicians and others during hospital disaster response.
